# A novel imaging index for predicting adverse progression in acute-on-chronic liver failure related to hepatitis B virus: the low erector spine index

**DOI:** 10.1186/s12876-023-02995-x

**Published:** 2023-10-26

**Authors:** Chao Zhou, Yuan Liu, Xiaoxiao Liang, Ning Zhang, Tingting He, Jingjing Zhang, Jin Zhang, Shuangnan Fu, Xin Li, Pengcheng Liu, Tianyi Zhang, Man Gong

**Affiliations:** 1 Department of Liver Disease, 5th Medical Center of Chinese PLA General Hospital, No. 100, the 4th Ring Road, Beijing, China; 2 Department of Diagnostic Radiology, 5th Medical Center of Chinese PLA General Hospital, No. 100, the 4th Ring Road, Beijing, China; 3 Beijing Chaoyang Integrative Medicine Emergency Center, Beijing, China

**Keywords:** Erector spine mass, Prognosis, Liver Failure, Hepatic encephalopathy, Kidney dysfunction

## Abstract

**Background:**

It is widely known that muscle mass influences the outcomes of many chronic diseases. Erector spine mass is a convenient parameter obtained from routine abdominal computed tomography (CT). The clinical application value of erector spine mass, and whether erector spine mass could predict the outcome of disease has not been studied.

**Aim:**

To evaluate the role of the erector spine index (ESI) calculated based on abdominal CT imaging in the progression of acute-on-chronic liver failure related to the hepatitis B virus (HBV-ACLF).

**Methods:**

We performed a retrospective study of 118 HBV-ACLF patients and calculated the ESI (the total erector spine area normalized for height^2^ in meters) for each patient through abdominal CT. The findings were analyzed regarding the progression of HBV-ACLF and the ESI at baseline, including mortality and the development of complications.

**Results:**

The ESI level was associated with mortality and the development of complications. During the 90-day follow-up period, patients with a low ESI (<12.05 cm^2^/m^2^) had higher mortality than those with a high ESI (≥ 12.05 cm^2^/m^2^) (51.7% vs. 26.7%), and the cumulative survival rates were 71.0%±4.6 and 85.8%±3.9, respectively (log-rank P = 0.003). The hazard ratios (HRs) calculated using univariable and multivariable analyses were 2.23(95% confidence interval (CI): 1.25–4.21, P = 0.005) and 2.52 (95% CI: 1.34–9.24, P = 0.011), respectively. Patients with a low ESI (<12.05 cm^2^/m^2^) had higher incidences of kidney dysfunction (43.5% vs. 23.2%, P = 0.029; log-rank P = 0.017) and hepatic encephalopathy (39.6% vs. 14.0%, P = 0.003; log-rank P = 0.010) than those with a high ESI. A low ESI was an independent risk factor for kidney dysfunction (adjusted HR = 1.36, 95% CI: 1.05–2.93, P = 0.043) and the development of hepatic encephalopathy (adjusted HR = 2.26; 95% CI: 2.05–3.13, P = 0.036). In addition, the presence of hepatic encephalopathy (the odds ratio (OR) = 2.26, 95% CI: 2.05–3.18, P = 0.006), spontaneous bacterial peritonitis (OR = 3.95, 95% CI: 1.01–5.46, P = 0.037), and kidney dysfunction (OR = 4.47, 95% CI: 1.02–9.64, P = 0.032) was independently associated with a low ESI in patients.

**Conclusion:**

A low ESI is an independent risk factor for mortality in patients with HBV-ACLF, as well as the development of kidney dysfunction and hepatic encephalopathy.

**Supplementary Information:**

The online version contains supplementary material available at 10.1186/s12876-023-02995-x.

## Introduction

Sarcopenia is commonly found among patients with cirrhosis, with a prevalence rate of roughly 29-37.5%, and is gaining increasing attention [1, 2]. Loss of muscle mass, as the phenotypic representation of sarcopenia, is disadvantageous for prognosis in these patients [3, 4]. This loss of muscle mass is associated with the development of acute-on-chronic liver failure (ACLF) and influences post-liver transplantation survival for ACLF patients [5–7]. Accurate methods to assess muscle mass in patients include bioelectrical impedance analysis and dual-energy X-ray absorptiometry, which are dependent on specialized equipment and are costly. Measures of muscle mass by cross-sectional imaging are reasonable and convenient because routine screening of computed tomography (CT) or magnetic resonance imaging (MRI) is necessary for patients with liver disease [8–10]. Commonly used measurement objects included the psoas area, psoas muscle thickness, lumbar muscle area located at the third lumbar vertebra (L3), and skeletal muscles of the extremities [11, 12]. Few studies have focused on erector spine mass, and growing evidence suggests that more indicators could be established for the comprehensive assessment of sarcopenia in cirrhotic patients [13]. We aimed to evaluate the role of erector spine mass located at the upper lumbar vertebra in the development of acute-on-chronic liver failure related to hepatitis B virus (HBV-ACLF) using erector spine area/height^2^.

## Materials and methods

### Study population

For this study, we included HBV-ACLF patients from the National Twelve Five-Year Science and Technology Major Project of China (ChiCTR-TRC-00000766), which prospectively enrolled HBV-ACLF patients in a follow-up program [14]. A prospective study was conducted from November 31, 2012, to December 31, 2014. All patients were recruited from the 5th Medical Center of Chinese PLA General Hospital. ACLF due to autoimmunity, drugs, alcohol, toxins, hepatocellular carcinoma, or parasites were excluded. The patients received treatment included nucleos(t)ide analogs, antibiotics, liver dialysis, or liver transplantation. Drawing from available data records, the primary endpoint was 90-day free-transplant mortality resulting from HBV-ACLF after enrollment and secondary endpoints were the development of complications, including overt hepatic encephalopathy, kidney dysfunction, and in-hospital infection, during follow-up. The patients who underwent abdominal cross-sectional imaging either 3 days prior or up to 3 days after enrollment and 90-day follow-up time were included. Patients who underwent liver dialysis or transplantation and those lacking key information or records were excluded from our study. Finally, 118 patients with HBV-ACLF were analyzed.

HBV-ACLF was defined as both ACLF and chronic liver disease due to hepatitis B virus infection. ACLF was defined according to the Asian Pacific Association for the Study of the Liver guideline [15]. The diagnostic criteria were (1) serum bilirubin ≥ 5 mg/dL, (2) international normalized ratio (INR) ≥ 1.5 or prothrombin activity ≤ 40%, and (3) ascites and/or encephalopathy as determined by physical examination. Chronic hepatitis B virus infection was diagnosed according to HBsAg positivity for more than 6 months.

Kidney dysfunction was defined as serum creatinine levels of 1.2 mg/dL or more [16]. The diagnosis of hepatic encephalopathy was made according to the West Haven classification. Spontaneous bacterial peritonitis (SBP) was defined as (1) ascites polymorphonuclear cell count ≥ 0.25 × 10^9^/L; (2) positive ascites bacterial culture; and (3) procalcitonin > 0.5 ng/mL, and infection of other sites was excluded [17]. In-hospital infection was defined as emerging infections regardless of the location identified 48 h after admission. Biochemical blood analyses were performed using standard laboratory tests.

### Image acquisition and assessment of muscle parameters

CT examinations were performed for regular screening or surveillance of hepatocellular carcinoma. Images were acquired with a 64-section CT scanner (GE, Lightspeed). Imaging parameters were as follows: non-contrast-enhanced; section thickness, 5 mm; 5 mm interval; tube voltage, 120 kV; and tube current, approximately 290 mA.

**Fig. 1:**
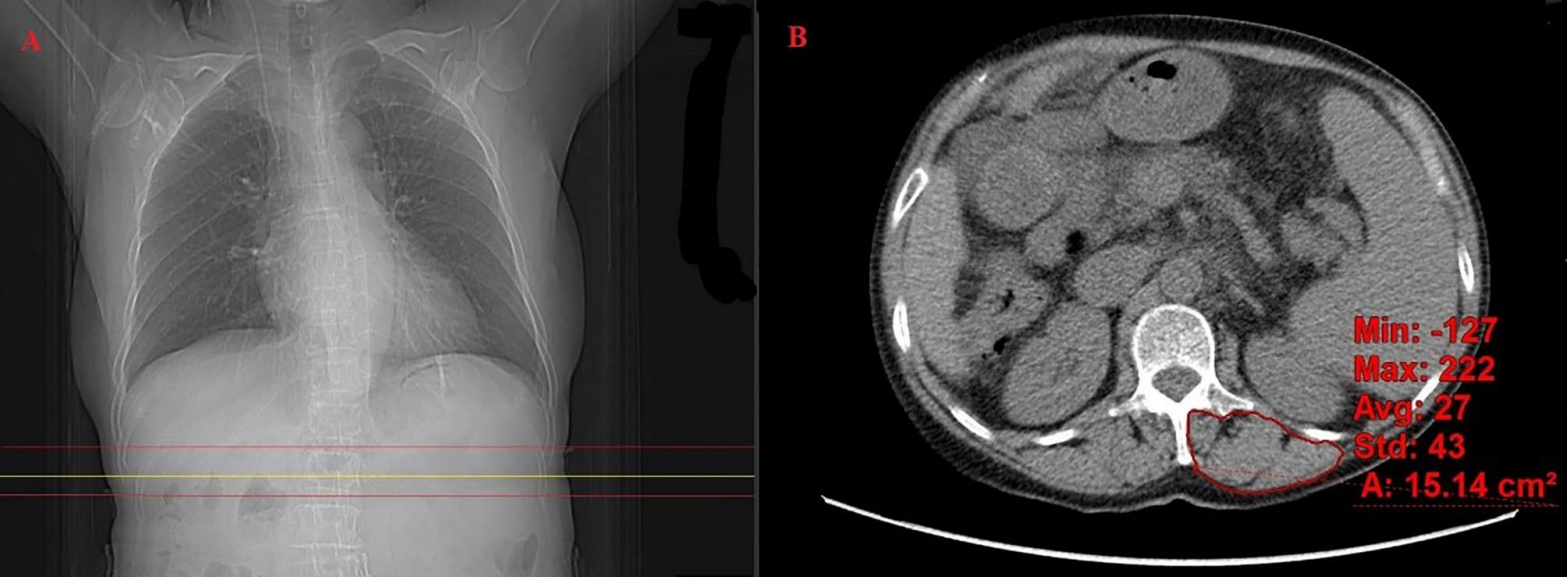
**(A)** CT in the coronal plane of a patient displaying the levels of muscle examination of different methods (yellow line: CT positioning line; upper red line: upper boundary at L1; lower red line: lower boundary at L1); **(B)** Exemplary CT of the cross-section of the erector spinal muscles in a patient. The image detail outlined by the red line depicts the detected erector spinal cross-sectional area. L1: the 1st lumbar vertebra

We analyzed the transversal erector spine index (ESI) and skeletal muscle index (SMI) at L3 in cross-sectional images. Using the CT positioning line function of the workstation, we located the corresponding axial level image according to the upper and lower boundaries at the 1st lumbar vertebra (L1) on the CT positioning images, manually outlined the complete range of the erector spine as the area of interest as the Hounsfield unit (HU) thresholds of − 29 to + 150 were used for the skeletal muscle [18], measured its area through the image workstation software (GE AW4.6), averaged the measurement 3 times to reduce the error, recorded the average value as the total erector spine area, and normalized for height^2^ (in meters) to calculate ESI (Fig. 1). The method used to calculate SMI was described in a previous study [19]. The assessments were performed independently by 2 hepatologists who had been trained by an expert radiologist. A third imaging physician was involved in reaching a consensus whenever disagreement arose.

### Statistics

Continuous variables were expressed as the mean ± standard deviation (SD) or medians and ranges, while categorical variables were expressed as frequencies and percentages. Univariable analyses included Student’s t-test for pairwise comparisons of parametric data distributions, the Mann–Whitney U test for pairwise comparisons of nonparametric distributions, and chi-square tests for comparisons of categorical variables. Binary logistic regression with forward elimination was used to evaluate independent factors with ESI. Kaplan–Meier curves were used to illustrate cumulative rates of survival, hepatic encephalopathy, kidney dysfunction, and in-hospital infection, and differences were assessed by the log-rank test. The association of risk factors with mortality and the development of complications was assessed by univariable and multivariable Cox proportional hazards regression models. The choice of variables for the multivariable analysis was based on the results of univariable analysis and clinical correlation. Cox proportional hazards regression was used for group comparisons of mortality. Pearson correlation analysis was used for the correlation of ESI with SMI. The area under the receiver operating characteristic curve (ROC) was plotted, and ROC analysis was performed based on the ESI and SMI. P < 0.05 was considered statistically significant for all tests. All statistical analyses were performed using IBM SPSS Statistics 20 (SPSS Inc.), and GraphPad Prism version 9 was used to create figures.

### Ethics

Our study was approved by the local institutional review board, and study procedures were performed following the Declaration of Helsinki’s ethical principles for medical research involving human subjects. Informed consent was obtained from all patients to be included in the study.

## Results

### General patient characteristics

In this study, 118 patients with HBV-ACLF were included. Of these patients, 104 (88.1%) were men. The average age was 41.3 ± 10.7 years. The average level of HBV DNA was 3.2 ± 2.2 log_10_ IU/ml. The average model for end-stage liver disease (MELD) score and MELD-sodium (MELD-Na) score were 27.4 ± 6.8 and 30.4 ± 9.4, respectively. The potential precipitating event included discontinuance of nucleos(t)ide analogs for 36 patients who had taken entecavir or adefovir combined with lamivudine, the resistance of nucleos(t)ide analogs for 9 patients who had taken lamivudine or telbivudine, and severe acute attack for 34 patients who had not previously received nucleos(t)ide analogs. There was no record of identified precipitating for 39 patients. All patients received entecavir (92.4%) or a combination of entecavir and adefovir (7.6%) after onset. The presence of ascites, SBP, kidney dysfunction, hyponatremia, respiratory infections, and hepatic encephalopathy at baseline was 89.8%, 21.2%, 13.5%, 42.4%, 14.4%, and 11.0%, respectively. Besides the infections located in the abdominal and pulmonary, infections involved in the urinary system, gastrointestinal tract, and unknown locations were 16.1%. The average ESI was 12.6 ± 3.8 cm^2^/m^2^. Laboratory parameters, such as serum albumin, bilirubin, creatinine, INR, and white blood cell count, were shown in detail in Table 1.

**Table 1:** General characteristics at baseline

Parameters	All patients(n = 118)	ESI (cm^2^/m^2^)				P value	
		<12.05 (n = 58)		≥ 12.05 (n = 60)			
*Demographic*							
Age (years)	42.9 ± 10.7	43.8 ± 11.8		42.2 ± 9.5		0.420	
Male, n (%)	104(88.1)	52(89.7)		52(86.7)		0.616	
ESI (cm^2^/m^2^)	12.6 ± 3.8	11.3 ± 4.0		13.5 ± 3.4		0.026	
*Laboratory indicators*							
Albumin (g/L)	30.2 ± 4.4	29.1 ± 5.1		32.2 ± 6.7		0.069	
Globulin (g/L)	25.9 ± 7.4	26.1 ± 8.1		25.7 ± 6.8		0.849	
Bilirubin (mg/dL)	20.1 ± 7.7	21.0 ± 8.3		19.2 ± 7.1		0.377	
ALT (U/L)	132(13-2329)	153(94–349)		117(38–496)		0.423	
AST (U/L)	166(47-1500)	208(134–349)		116(77–400)		0.243	
GGT (U/L)	64(15–231)	63(45–95)		68(37–103)		0.846	
Creatinine (µmol/L)	97.1 ± 17.0	98.6 ± 19.8		95.7 ± 14.3		0.526	
INR	3.1 ± 0.5	2.1 ± 0.4		4.1 ± 0.6		0.172	
WBC (×10^9^/L)	7.0 ± 4.9	7.9 ± 6.4		6.2 ± 2.8		0.186	
Hemoglobin (g/L)	119 ± 23	117 ± 24		120 ± 22		0.564	
Platelet count (×10^9^/L)	85 ± 42	76 ± 33		93 ± 48		0.125	
Serum ammonia (mmol/L)	65.8 ± 22.4	69.1 ± 25.8		62.4 ± 18.2		0.030	
HBV DNA (log_10_ IU/ml)	3.2 ± 2.2	3.0 ± 2.1		3.3 ± 2.3		0.643	
MELD	27.4 ± 6.8	28.2 ± 8.7		26.6 ± 3.7		0.365	
MELD-Na	30.4 ± 9.4	30.7 ± 7.9		30.2 ± 10.8		0.837	
*Complications at baseline*							
Ascites, n (%)	106(89.8)	54(93.1)		52(86.7)		0.247	
SBP, n (%)	25(21.2)	18(31.0)		7(11.7)		0.010	
Kidney dysfunction, n (%)	16(13.5)	12(20.7)		4(6.7)		0.026	
Hepatic encephalopathy, n (%)	13(11.0)	10(17.2)		3(5.0)		0.034	
Respiratory infections, n (%)	17(14.4)	9(15.5)		8(13.3)		0.736	
Hyponatremia, n (%)	50(42.4)	26(44.8)		24(40.0)		0.596	
Other types of infection, n (%)	19(16.1)	10(17.2)		9(15.0)		0.741	

Abbreviations. ESI: erector spine index, ALT: alanine transaminase, AST: aspartate transaminase, GGT: γ-glutamyl transferase, INR: international normalized ratio, WBC: white blood cell count, MELD: model for end-stage liver disease, MELD-Na: MELD-Sodium, SBP: spontaneous bacterial peritonitis

All patients were followed up to 90 days after enrollment. The free-transplant mortality was 39.0%, and the rates of new-onset kidney dysfunction, hepatic encephalopathy, and in-hospital infection were 32.4%, 25.7%, and 68.8%, respectively (Table 2). The in-hospital infections primarily included SBP, respiratory infections, and other types of infections, and the details were shown (Supplementary Table 1). Other types of infections in this study referred to conditions beyond those of abdominal and pulmonary and involved the urinary and gastrointestinal systems, as well as unlocated infections.

**Table 2:** Outcomes for 90-day follow up

Outcomes	All patients(n = 118)	ESI (cm^2^/m^2^)		P value
		<12.05 (n = 58)	≥ 12.05 (n = 60)	
Mortality, n (%)	46(39.0)	30(51.7)	16(26.7)	0.005
Development of kidney dysfunction, n (%)^a^	33(32.4)	20(43.5)	13(23.2)	0.029
Development of hepatic encephalopathy, n (%)^b^	27(25.7)	19(39.6)	8(14.0)	0.003
Development of in-hospital infection, n (%)^c^	53(68.8)	27(75.0)	26(63.4)	0.273

Abbreviations. ESI: erector spine index. a: Except for 16 patients with kidney dysfunction at baseline including 12 in the group with ESI<12.05 cm^2^/m^2^ and 4 in the group with ESI ≥ 12.05 cm^2^/m^2^; b: Except for 13 patients with hepatic encephalopathy at baseline including 10 in group with ESI<12.05 cm^2^/m^2^ and 3 in group with ESI ≥ 12.05 cm^2^/m^2^; c: Except for 41 patients with in-hospital infection at baseline including 22 in group with ESI<12.05 cm^2^/m^2^ and 19 in group with ESI ≥ 12.05 cm^2^/m^2^

### Correlation of ESI with baseline characteristics

According to the median of ESI (12.05 cm^2^/m^2^), the patients were classified into two groups to evaluate the factors associated with erector spine mass in HBV-ACLF patients. The cut-off value of ESI calculated through ROC was 12.06 cm^2^/m^2^, which closely approximated the median. The baseline characteristics between the two groups were compared in Table 1. Patients with ESI<12.05 cm^2^/m^2^ (n = 58) showed a higher level of serum ammonia (P = 0.030) than those patients with ESI ≥ 12.05 cm^2^/m^2^ (n = 60). There was no significant difference in age, MELD score, MELD-Na score, or other laboratory indicators. Further binary logistic regression analysis was used to determine factors independently associated with ESI (Table 3). The presence of hepatic encephalopathy (the odds ratio (OR) = 2.26, 95% confidence interval (CI): 2.05–3.18; P = 0.006), SBP (OR = 3.95, 95% CI: 1.01–5.46; P = 0.037), and kidney dysfunction (OR = 4.47, 95% CI: 1.02–9.64; P = 0.032) were independently associated with low ESI in HBV-ACLF patients.

**Table 3:** Risk factors associated with ESI in HBV-ACLF patients

Parameters	Univariate analysis					Multivariate analysis			
	OR	95% CI	P value			OR		95% CI	P value
Age, per year	1.01	0.97–1.07	0.566						
Gender (male vs. female^‡^)	1.33	0.27–6.56	0.723						
Bilirubin, per 1 mg/dl	1.03	0.96–1.11	0.372						
Albumin, per 1 g/L	0.89	0.78–1.01	0.076						
Creatinine, per 1 µmol/L	1.01	0.98–1.04	0.520						
INR, per 1 unit	0.84	0.48–1.47	0.543						
ALT, per 1 U/L	1.00	1.00–1.00	0.223						
AST, per 1 U/L	1.00	1.00–1.00	0.597						
GGT, per 1 U/L	1.00	0.99–1.01	0.673						
Hemoglobin, per 1 g/L	0.99	0.97–1.02	0.557						
Serum Ammonia, per 1 mmol/L	0.99	0.96–1.01	0.986						
Hepatic encephalopathy (yes vs.no^‡^)	4.17	1.15–5.04	0.029			2.26		2.05–3.18	0.006
Sepsis (yes vs.no^‡^)	0.90	0.30–2.69	0.850						
SBP (yes vs.no^‡^)	2.87	0.99–8.37	0.053			3.95		1.01–5.46	0.037
Ascites (yes vs.no^‡^)	1.60	0.61–2.24	0.127						
Respiratory infections (yes vs.no^‡^)	1.24	0.40–3.83	0.711						
Kidney dysfunction (yes vs.no^‡^)	3.11	1.06–9.18	0.040			4.47		1.02–9.64	0.032
MELD, per 1 unit	1.01	0.98–1.23	0.794						
MELD-Na, per 1 unit	1.01	0.95–1.06	0.835						

^‡^: reference value. Abbreviations. OR: odds ratio, CI: confidence interval, INR: international normalized ratio, ALT: alanine transaminase, AST: aspartate transaminase, GGT: γ-glutamyl transferase, HE: hepatic encephalopathy, SBP: spontaneous bacterial peritonitis, MELD: model for end-stage liver disease, MELD-Na: MELD-Sodium.

### Impact of ESI on the free-transplant mortality of HBV-ACLF patients

**Fig. 2:**
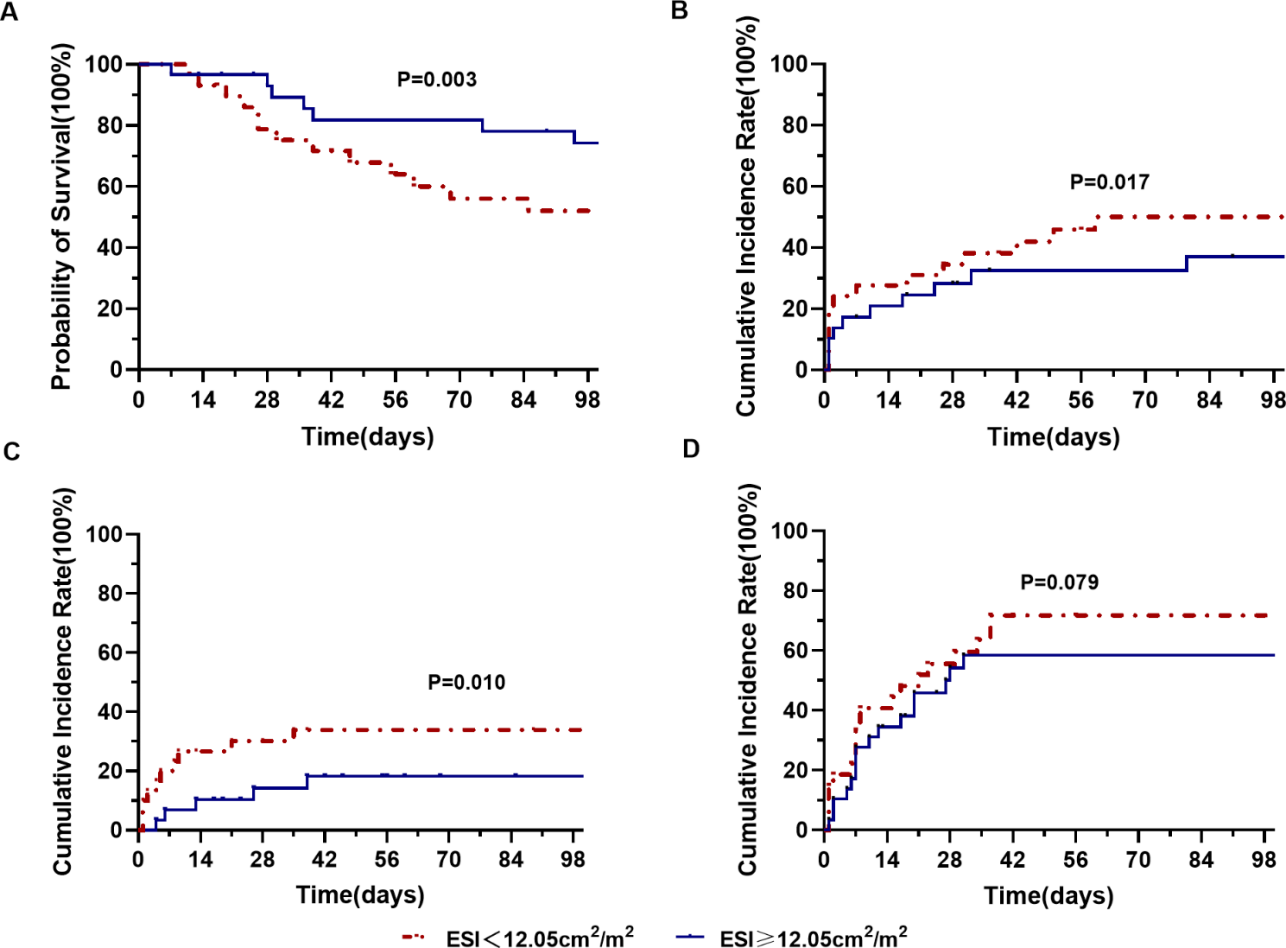
Kaplan–Meier curves of patients with a low ESI and patients with a high ESI level. Statistical significance was calculated by the log-rank test for **(A)** cumulative survival, **(B)** cumulative incidence rate of kidney dysfunction, **(C)** cumulative incidence rate of hepatic encephalopathy, and **(D)** cumulative incidence rate of in-hospital infection

During the 90-day follow-up period, 30 of 58 (51.7%) patients with a low ESI and 16 of 60 (26.7%) patients with a high ESI died(P = 0.005) (Table 2). Kaplan-Meier survival analysis was conducted, and the cumulative survival rates were 71.0%±4.6 and 85.8%±3.9, respectively (log-rank P = 0.003) (Fig. 2A). The hazard ratio (HR) calculated using univariable analysis was 2.23(95% CI: 1.25–4.21; P = 0.005) and further multivariable analyses showed that a low ESI increased the risk for death dependently (adjusted HR = 2.52; 95% CI: 1.34–9.24; P = 0.011) (Table 4). The details about the comparison between the survival patients with the deceased patients and the multiple analysis were provided in Supplementary Table 2 and Table 1, respectively.

**Table 4:** Cox Regression Evaluation of ESI Associated with Outcomes in HBV-ACLF patients

Outcomes	ESI (cm^2^/m^2^)	No. of events (%)	Univariate analysis			Multivariate analysis^*^	
			HR (95% CI)	P value		HR (95% CI)	P value
Mortality	ESI<12.05	30(51.7)	2.23(1.25–4.21)	0.005		2.52(1.34–9.24) ^d^	0.011
	ESI ≥ 12.05^‡^	16(26.7)	1.00			1.00	
Kidney dysfunction	ESI<12.05	20(43.5)	1.47(1.01–2.15)	0.017		1.36(1.05–2.93) ^e^	0.043
	ESI ≥ 12.05^‡^	13(23.2)	1.00			1.00	
Hepatic encephalopathy	ESI<12.05	19(39.6)	2.54(1.21–5.31)	0.014		2.26(2.05–3.13) ^f^	0.036
	ESI ≥ 12.05^‡^	8(14.0)	1.00			1.00	
In-hospital infection	ESI<12.05	27(75.0)	1.47(0.94–2.31)	0.090		1.62(0.6–3.08) ^g^	0.138
	ESI ≥ 12.05^‡^	26(63.4)	1.00			1.00	

^‡^: reference value. *: The choice of variables for the multivariable analysis was based on the results of univariable analysis and clinical correlation. d: HR adjusted by age, serum bilirubin, INR, hepatic encephalopathy, hyponatremia, and kidney dysfunction; e: HR adjusted by age, serum albumin, serum bilirubin; f: HR adjusted by serum albumin, serum bilirubin, INR, serum ammonia; g: HR adjusted by age, serum albumin, serum bilirubin, INR, presence of ascites at baseline. Abbreviations. ESI: erector spine index, HR: hazard ratio, CI: confidence interval, INR: international normalized ratio

### Impact of ESI on the development of kidney dysfunction, hepatic encephalopathy, and in-hospital infection

In the study, there were 102 patients with the absence of kidney dysfunction at baseline. Among these patients, 20 of 46 (43.5%) patients with a low ESI and 13 of 56 (23.2%) patients with a high ESI developed kidney dysfunction during the 90-day follow-up period(P = 0.029) (Table 2). There was a significant difference in the cumulative incidence rate of kidney dysfunction between groups (log-rank P = 0.017) (Fig. 2B). Univariable and multivariable analyses are shown in Table 4, indicating that a low ESI was an independent risk factor for kidney dysfunction (adjusted HR = 1.36; 95% CI: 1.05, 2.93; P = 0.043) (Table 4, and Supplementary Table 4 for details).

Furthermore, among 105 patients without the presence of hepatic encephalopathy at baseline, 49 patients with a low ESI had a higher cumulative incidence rate of hepatic encephalopathy compared with 56 patients with a high ESI as shown by Kaplan-Meier analysis (39.6% vs. 14.0%, P = 0.003; log-rank P = 0. 010) (Table 2; Fig. 2C), and a low ESI was an independent risk factor for the development of hepatic encephalopathy through multivariable analyses (adjusted HR = 2.26; 95% CI: 2.05, 3.13; P = 0.036) (Table 4, and Supplementary Table 5 for details). However, there was no difference in the cumulative incidence rate of in-hospital infection between the two groups (75.0% vs. 63.4%, P = 0.273; log-rank P = 0. 079; adjusted HR = 1.62; 95% CI: 0.60, 3.08; P = 0.138) (Fig. 2D; Table 4, and Supplementary Table 6 for details).

### Correlation of ESI with SMI

**Fig. 3:**
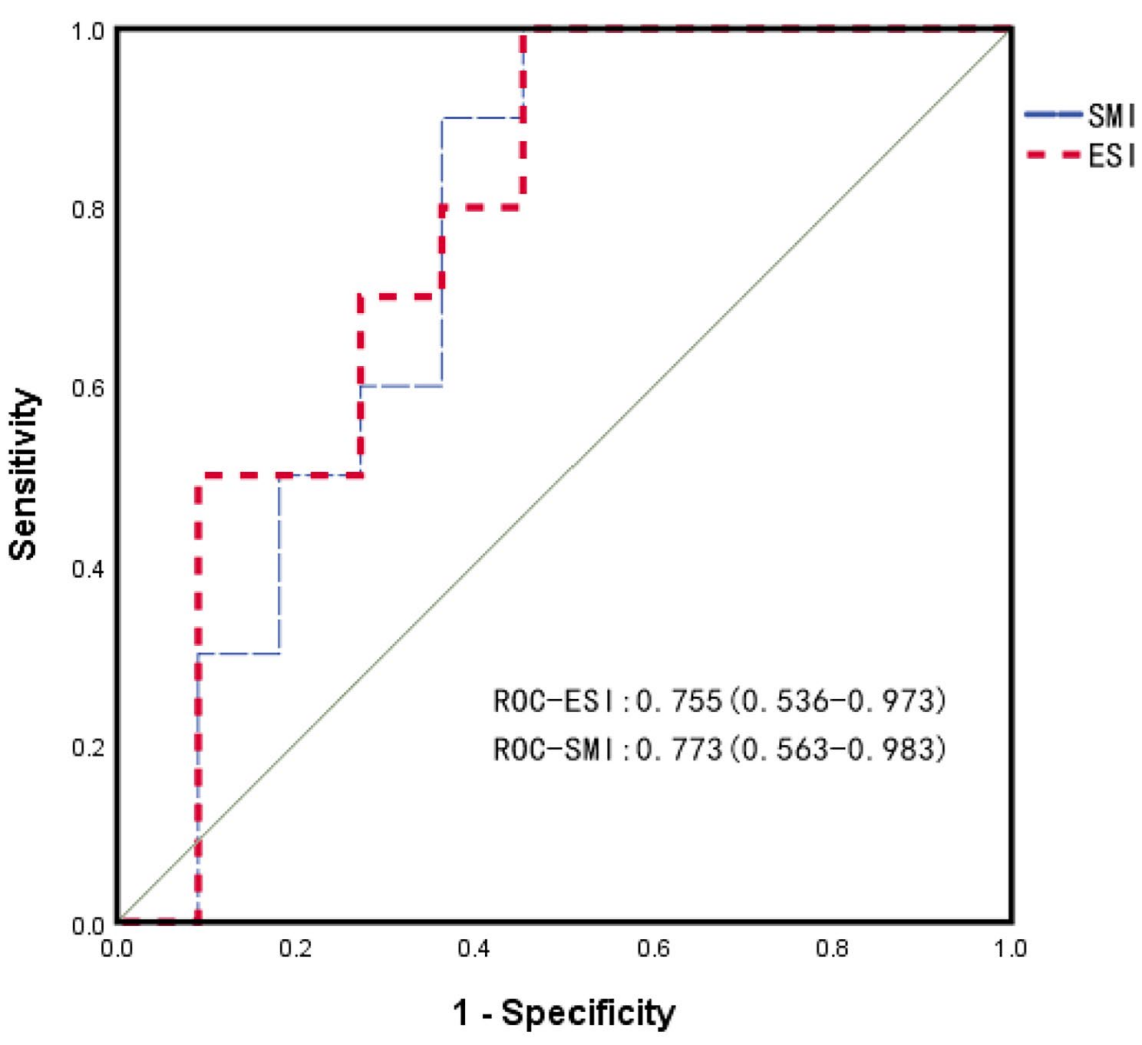
ROC for evaluating mortality in patients with HBV-ACLF. The areas under the ROC (95% CI) of the ESI and SMI were 0.755 (0.536–0.973) and 0.773 (0.563–0.983), respectively. ESI: erector spine index. SMI: skeletal muscle index

In this study, there were 23 patients with whole abdominal CT images. The ESI and SMI at L3 of these patients were calculated. The average ESI and SMI were 11.7 ± 3.8 and 39.4 ± 12.3, respectively (P = 0.231). There was a significant positive correlation between ESI and SMI (r = 0.714, P < 0.001). We also assessed the predictive ability of ESI and SMI for mortality outcomes. The areas under the ROC of ESI and SMI were 0.755 (0.536–0.973) and 0.773 (0.563–0.983), respectively (Fig. 3). The ROC analysis yielded no significant results (Z=-1.449, P = 0.147).

## Discussion

Previous research has suggested that sarcopenia was considered a clinical phenotype of malnutrition in cirrhosis [8], which represents the loss of muscle mass. The prevailing perspective appears to have advanced beyond the aforementioned statement. Due to the inconvenience of bedside assessment tools, obtaining cross-sectional muscle mass measurements from scheduled abdominal CT imaging, which is planned for screening hepatic cell carcinoma, has become the overwhelming choice, and the skeletal muscle indices at L3 are commonly adopted. However, routine CT imaging of the liver is positioned from the top of the diaphragm to the inferior margin of the liver (approximately L1). A rough count of routine CT imaging of the liver performed during one month at our institution showed that approximately 85.3% (1749/2050) did not effectively acquire the images of the psoas muscle at L3, while the erector spine could be acquired at the upper lumbar vertebra for all CT imaging of the liver. Therefore, without additional scanning, which could increase exposure to ionizing radiation for patients, we aimed to evaluate the association between CT-based erector spine mass indicators at baseline and the progression of HBV-ACLF in this study.

In our study, an association between ESI and free-transplant mortality was assessed in HBV-ACLF patients. In patients with relatively low ESI, the free-transplant mortality increased. We found that low levels of ESI were independent risk factors increasing the poor prognosis of HBV-ACLF, and HBV-ACLF patients with a low ESI had more than twice the risk of death as those with a high ESI. These results indicated that erector spine mass loss increased HBV-ACLF mortality. On the other hand, we found that a low ESI was associated with higher rates of overt hepatic encephalopathy and high serum ammonia than at baseline. Furthermore, the development of hepatic encephalopathy also increased in patients with a low ESI during follow-up. Apart from the association with the presence of hepatic encephalopathy, patients with a low ESI had a high risk for the development of kidney dysfunction. However, erector spine loss was not reflected in the MELD score or MELD-Na score. The lack of significant difference in erector spine loss between males and females in our study may be related to the small number of females involved and the limited sample size. There are no studies describing the distribution pattern of the erector spine in the population, and the differences in performance by gender require further research. Our study showed a significant correlation of ESI with SMI, therefore, low ESI could be considered as an index that reflected loss of skeletal muscle mass or sarcopenia in HBV-ACLF patients.

It has been reported that sarcopenia in patients with cirrhosis adversely affects clinical outcomes, including the overall risk of death, sepsis-related mortality, worse health-related quality of life [20], and development of hepatic decompensation, such as hepatic encephalopathy and infection [4, 21, 22]. In addition, it also impacted mortality related to treatment in patients with hepatic cell carcinoma [23], significant liver fibrosis [24], and adverse posttransplant outcomes [25, 26]. Our results were similar to previous studies, and loss of erector spine mass was considered as a risk factor related to poor outcomes and a high prevalence of complications in HBV-ACLF patients. Loss of skeletal muscle mass has impacts on reduced quality of life and longer hospital stays [20, 27]. Muscle depletion could reduce the capacity for extrahepatic ammonia removal [28, 29]. These factors may contribute to the deterioration of the disease. The pathophysiological basis of the impact of the loss of skeletal muscle mass on the course of HBV-ACLF requires further evaluation.

Previous studies have demonstrated that some pathways related to chronic liver disease and cirrhosis could contribute to sarcopenia, including an imbalance between energy needs and intake, reduced levels of circulating branched-chain amino acids leading to accelerated muscle breakdown, myotoxicity associated with systemic ammonia, dysbiosis, and disruption of mediators of the “liver muscle axis” [21–23]. There are potential strategies to improve sarcopenia, including specific amino acid supplementation, mitochondrial protection, and combination endurance-resistance exercise [30–33]. Transjugular intrahepatic portosystemic shunt placement was confirmed to improve skeletal muscle and fat mass in cirrhotic patients with sarcopenia, and the reversal of sarcopenia could reduce the risk of death [19]. Future trials are still needed to confirm the impact of sarcopenia reversal on clinical outcomes.

Our study mainly demonstrated the impact of skeletal muscle loss on the progression of HBV-ACLF. The highlight of this study is the finding of the effect of the ESI as an index associated with muscle reduction on the mortality of HBV-ACLF and the development of complications. This study has some limitations. First, this study was designed retrospectively to explore the impact of ESI on the progression of HBV-ACLF, and it was dependent on data collected previously. Some potential factors were missing, such as diabetes, direct causes of death, and infection-related pathogen testing. We employed multivariate analysis to account for most of the potential confounding as much as feasible. Second, we only analyzed the correlation and predictive ability of ESI with SMI for a minority of patients, and the collection of these data involving larger populations would allow for a more comprehensive assessment of ESI for the diagnosis of sarcopenia and predicting disease progression in HBV-ACLF patients.

In conclusion, our study showed that erector spine mass was significantly associated with the outcomes of HBV-ACLF. The risk of death, development of hepatic encephalopathy, and kidney dysfunction increased in HBV-ACLF patients with a low ESI. Therefore, HBV-ACLF patients with low ESI need strengthened monitoring due to the high risk of death and disease progression.

### Electronic supplementary material

Below is the link to the electronic supplementary material.

Supplementary Material 1

## Data Availability

The datasets used and/or analyzed during the current study are available from the corresponding author upon reasonable request.

## Abbreviations

ACLF: Acute-on-chronic liver failure
ALT: Alanine transaminase
AST: Aspartate transaminase
CI: Confidence interval
CT: Computed tomography
ESI: Erector spine index
GGT: γ-glutamyl transferase
HBV-ACLF: Acute-on-chronic liver failure related to the hepatitis B virus
HR: Hazard ratio
HU: Hounsfield unit
INR: International normalized ratio
L1: The 1st lumbar vertebra
L3: The third lumbar vertebra
MELD: Model for end-stage liver disease
MELD-Na: MELD-sodium
MRI: Magnetic resonance imaging
OR: Odds ratio
SBP: Spontaneous bacterial peritonitis
SMI: Skeletal muscle index
SD: Standard deviation
