# Supplementary material for: A novel imaging index for predicting adverse progression in acute-on-chronic liver failure related to hepatitis B virus: the low erector spine index
Source: BMC Gastroenterol. 2023 Oct 26;23:367. doi: 10.1186/s12876-023-02995-x (PMC10601125; doi:10.1186/s12876-023-02995-x)
Supplement: Supplementary file 1 — Supplementary Material 1 [file 12876_2023_2995_MOESM1_ESM.docx]

**Supplementary Table 1 The types of in-hospital infection**

| **ESI (cm^2^/m^2^)** | **Types of in-hospital infection(n=77)** | | | **P value** |
| --- | --- | --- | --- | --- |
|  | **Spontaneous bacterial peritonitis, n (%)** | **Respiratory infections, n (%)** | **Other types of infections, n (%)** |  |
| **＜12.05 (n=36)** | 16(44.4%) | 15(41.7%) | 5(13.9%) | 0.299 |
| **≥12.05 (n=41)** | 20(48.8%) | 11(26.8%) | 10(24.4%) |  |

Abbreviations. ESI: erector spine index.

**Supplementary Table 2 Comparisons of Deceased and** **Surviving Patients**

| **Parameters** | **Deceased patients（n=46）** | **Surviving patients (n=72)** | **P value** |
| --- | --- | --- | --- |
| Age (years) | 45.3±8.5 | 41.5±11.6 | 0.059 |
| Male, n (%) | 40(87.0) | 64(88.9) | 0.752 |
| ESI, cm^2^/m^2^ | 9.4±1.6 | 15.7±2.4 | ＜0.001 |
| Bilirubin (mg/dL) | 23.1±7.8 | 18.1±6.9 | ＜0.001 |
| Creatinine (μmol/L) | 100.9±19.9 | 94.6±14.4 | 0.051 |
| INR | 3.6±0.6 | 2.7±0.6 | 0.046 |
| WBC (×10^9^/L) | 7.8±5.8 | 6.5±4.2 | 0.165 |
| Hemoglobin (g/L) | 114±21 | 122±24 | 0.083 |
| Platelet count (×10^9^/L) | 76±41 | 90±42 | 0.099 |
| MELD | 29.4±8.1 | 26.1±5.4 | 0.009 |
| MELD-Na | 35.1±11.0 | 27.3±6.4 | ＜0.001 |
| HE, n (%) | 9(19.6) | 4(5.6) | 0.018 |
| SBP, n (%) | 13(28.3) | 12(16.7) | 0.133 |
| Respiratory infections, n (%) | 10(21.7) | 7(9.7) | 0.070 |
| Other infection, n (%) | 9(19.6) | 10(13.9) | 0.413 |
| Hyponatremia, n (%) | 29(63.0) | 21(30.0) | ＜0.001 |
| Ascites, n (%) | 46(100.0) | 60(83.3) | 0.009 |
| Kidney dysfunction, n (%) | 10(21.7) | 6(8.3) | 0.038 |

Abbreviations. ESI: erector spine index, INR: international normalized ratio, WBC: white blood cell count, MELD: model for end-stage liver disease, MELD-Na: MELD-Sodium, HE: hepatic encephalopathy, SBP: spontaneous bacterial peritonitis.

**Supplementary Table 3 The impact of ESI on the mortality of HBV-ACLF patients**

| **Parameters** | **Univariate Cox regression** | | |  | | **Multivariate Cox regression** | | |
| --- | --- | --- | --- | --- | --- | --- | --- | --- |
|  | **HR** | **95% CI** | **P value** | |  | **HR** | **95% CI** | **P value** |
| ESI(＜12.05 vs. ≥12.05^╪^) | 2.23 | 1.25-4.21 | 0.005 | | | 2.52 | 1.34-9.24 | 0.011 |
| Age, per year | 1.03 | 1.02-1.07 | 0.026 | | | 1.03 | 0.99-1.06 | 0.081 |
| Bilirubin, per 1 mg/dl | 1.07 | 1.02-1.12 | <0.001 | | | 1.04 | 1.00-1.08 | 0.037 |
| INR, per 1 unit | 1.09 | 1.05-1.14 | <0.001 | | | 1.06 | 1.02-1.10 | 0.005 |
| HE (yes vs.no^╪^) | 2.32 | 1.12-4.82 | 0.024 | | | 3.26 | 1.35-7.93 | 0.009 |
| Hyponatremia (yes vs.no^╪^) | 2.74 | 1.50-4.99 | 0.001 | | | 1.82 | 0.91-3.63 | 0.091 |
| Kidney dysfunction (yes vs.no^╪^) | 2.29 | 1.13-4.61 | 0.021 | | | 1.61 | 0.74-3.52 | 0.228 |
| WBC, per 1×10^9^/L | 1.05 | 0.99-1.10 | 0.064 | | | - | - | - |
| Platelet count, per 1×10^9^/L | 0.99 | 0.98-1.01 | 0.127 | | | - | - | - |
| SBP (yes vs.no^╪^) | 1.63 | 0.86-3.10 | 0.136 | | | - | - | - |
| Respiratory infections (yes vs.no^╪^) | 1.71 | 0.85-3.45 | 0.134 | | | - | - | - |
| Other infections (yes vs.no^╪^) | 1.36 | 0.66-2.82 | 0.406 | | | - | - | - |
| Ascites (yes vs.no^╪^) | 5.61 | 0.68-99.86 | 0.079 | | | - | - | - |

Abbreviations. ESI: erector spine index, INR: international normalized ratio, HE: hepatic encephalopathy, WBC: white blood cell count, SBP: spontaneous bacterial peritonitis.

**Supplementary Table 4 The impact of ESI on the development of kidney dysfunction for HBV-ACLF patients**

| **Parameters** | **Univariate Cox regression** | | |  | | **Multivariate Cox regression** | | |
| --- | --- | --- | --- | --- | --- | --- | --- | --- |
|  | **HR** | **95% CI** | **P value** | |  | **HR** | **95% CI** | **P value** |
| ESI(＜12.05 vs. ≥12.05^╪^) | 1.47 | 1.01-2.15 | 0.017 | | | 1.36 | 1.05-2.93 | 0.043 |
| Age, per year | 1.06 | 1.02-1.13 | 0.010 | | | 1.08 | 1.02-1.15 | 0.008 |
| Albumin, per 1 g/L | 0.94 | 0.39-0.98 | 0.007 | | | 0.81 | 0.63-0.97 | 0.004 |
| Bilirubin, per 1 mg/dl | 1.07 | 1.02-1.12 | 0.010 | | | 1.28 | 1.01-1.62 | 0.044 |
| INR, per 1 unit | 1.01 | 0.92-1.10 | 0.901 | | | - | - | - |
| HE (yes vs.no^╪^) | 3.78 | 0.52-7.45 | 0.189 | | | - | - | - |
| WBC, per 1×10^9^/L | 1.06 | 0.99-1.14 | 0.061 | | | - | - | - |
| Platelet count, per 1×10^9^/L | 0.91 | 0.99-1.02 | 0.253 | | | - | - | - |
| SBP (yes vs.no^╪^) | 1.63 | 0.39-2.28 | 0.897 | | | - | - | - |
| Respiratory infections (yes vs.no^╪^) | 1.30 | 0.29-5.88 | 0.731 | | | - | - | - |
| Other infections (yes vs.no^╪^) | 1.30 | 0.12-1.89 | 0.293 | | | - | - | - |
| Ascites (yes vs.no^╪^) | 1.06 | 0.37-3.08 | 0.912 | | | - | - | - |

Abbreviations. ESI: erector spine index, INR: international normalized ratio, HE: hepatic encephalopathy, WBC: white blood cell count, SBP: spontaneous bacterial peritonitis.

**Supplementary Table 5 The impact of ESI on the development of hepatic encephalopathy (HE) for HBV-ACLF patients**

| **Parameters** | **Univariate Cox regression** | | |  | | **Multivariate Cox regression** | | |
| --- | --- | --- | --- | --- | --- | --- | --- | --- |
|  | **HR** | **95% CI** | **P value** | |  | **HR** | **95% CI** | **P value** |
| ESI(＜12.05 vs. ≥12.05^╪^) | 2.54 | 1.21-5.31 | 0.014 | | | 2.26 | 2.05-3.13 | 0.036 |
| Albumin, per 1 g/L | 0.93 | 0.83-0.99 | 0.031 | | | 0.87 | 0.77-0.99 | 0.033 |
| Bilirubin, per 1 mg/dl | 1.07 | 1.02-1.12 | 0.010 | | | 1.09 | 1.00-1.20 | 0.050 |
| INR, per 1 unit | 1.13 | 1.06-1.21 | <0.001 | | | 1.10 | 1.05-1.14 | 0.036 |
| Serum ammonia, per 1mmol/L | 1.23 | 1.01-1.25 | 0.027 | | | 1.61 | 0.45-5.76 | 0.462 |
| Age, per year | 1.00 | 0.95-1.05 | 0.955 | | | - | - | - |
| WBC, per 1×10^9^/L | 1.09 | 1.01-1.17 | 0.055 | | | - | - | - |
| Platelet count, per 1×10^9^/L | 0.93 | 0.55-1.07 | 0.116 | | | - | - | - |
| SBP (yes vs.no^╪^) | 1.28 | 0.92-1.77 | 0.140 | | | - | - | - |
| Respiratory infections (yes vs.no^╪^) | 1.24 | 0.13-1.46 | 0.851 | | | - | - | - |
| Other infections (yes vs.no^╪^) | 2.71 | 0.51-4.40 | 0.243 | | | - | - | - |
| Ascites (yes vs.no^╪^) | 1.62 | 0.42-6.20 | 0.484 | | | - | - | - |
| Kidney dysfunction (yes vs.no^╪^) | 1.96 | 0.45-7.15 | 0.387 | | | - | - | - |

Abbreviations. ESI: erector spine index, INR: international normalized ratio, WBC: white blood cell count, SBP: spontaneous bacterial peritonitis.

**Supplementary Table 6 The impact of ESI on the development of in-hospital infection for HBV-ACLF patients**

| **Parameters** | **Univariate Cox regression** | | |  | | **Multivariate Cox regression** | | |
| --- | --- | --- | --- | --- | --- | --- | --- | --- |
|  | **HR** | **95% CI** | **P value** | |  | **HR** | **95% CI** | **P value** |
| ESI (＜12.05 vs. ≥12.05^╪^) | 1.47 | 0.94-2.31 | 0.090 | | | 1.62 | 0.6-3.08 | 0.138 |
| Age, per year | 1.03 | 1.02-1.07 | 0.038 | | | 1.02 | 0.96-1.04 | 0.905 |
| Albumin, per 1 g/L | 0.98 | 0.96-0.99 | 0.034 | | | 0.99 | 0.98-1.01 | 0.302 |
| Bilirubin, per 1 mg/dl | 1.07 | 1.02-1.12 | 0.010 | | | 1.05 | 0.99-1.10 | 0.066 |
| INR, per 1 unit | 1.08 | 1.01-1.16 | 0.027 | | | 1.63 | 1.16-4.07 | 0.016 |
| Ascites (yes vs.no^╪^) | 1.77 | 1.01-2.27 | 0.024 | | | 1.41 | 0.97-1.84 | 0.193 |
| WBC, per 1×10^9^/L | 1.04 | 0.98-1.96 | 0.093 | | | - | - | - |
| Platelet count, per 1×10^9^/L | 0.99 | 0.98-1.01 | 0.245 | | | - | - | - |
| HE (yes vs.no^╪^) | 2.34 | 0.69-6.07 | 0.131 | | | - | - | - |
| Kidney dysfunction (yes vs.no^╪^) | 1.13 | 0.91-1.41 | 0.256 | | | - | - | - |

Abbreviations. ESI: erector spine index, INR: international normalized ratio, WBC: white blood cell count, HE: hepatic encephalopathy.
